# COVID-19 Pandemic: Impact of Quarantine on Medical Students’ Mental Wellbeing and Learning Behaviors

**DOI:** 10.12669/pjms.36.COVID19-S4.2809

**Published:** 2020-05

**Authors:** Sultan Ayoub Meo, Abdulelah Adnan Abukhalaf, Ali Abdullah Alomar, Kamran Sattar, David C Klonoff

**Affiliations:** 1 Sultan Ayoub Meo Department of Physiology, College of Medicine, King Saud University, Riyadh, Saudi Arabia; 2 Abdulelah Adnan Abukhalaf Department of Physiology, College of Medicine, King Saud University, Riyadh, Saudi Arabia; 3 Ali Abdullah Alomar Department of Physiology, College of Medicine, King Saud University, Riyadh, Saudi Arabia; 4 Kamran Sattar Department of Medical Education, College of Medicine, King Saud University, Riyadh, Saudi Arabia; 5 David C Klonoff Diabetes Research Institute, Mills-Peninsula Medical Center San Mateo, California, USA

**Keywords:** COVID 19, Quarantine, Mental Wellbeing, Learning Behaviors

## Abstract

**Background and Objectives:**

The novel coronavirus COVID-19 pandemic causes great public health and socioeconomic harms. Worldwide many countries implemented quarantine policies to minimize the spread of this highly contagious disease. The present study aim was to investigate the impact of quarantine on the medical students’ mental wellbeing and learning behaviors.

**Methods:**

In this descriptive study, we used a questionnaire with a Five-Point Likert Scale to collect the information. The questionnaire was distributed among 625 medical students through their emails with a response rate of 530 (84.8%), majority 294 (55.47%) being female. The survey questionnaire consisted of total 20 items; 12 items were related to psychological wellbeing and stress-allied queries and 08 items were about learning behaviors.

**Results:**

The findings encompass two important characteristics related to quarantine, psychological wellbeing, and learning behaviors. A combined cohort of 234 medical students, either female or male, (which was 44.1% of the total responders) showed a sense of being emotionally detached from family, friends and fellow students, 125/ 530 (23.5%) medical students felt disheartened. Both female and male medical students showed a marked decrease in their overall work performance. Moreover, 56.2% of the total students (61.5% of the females and 49.5% of the males) felt a decrease in the time they spent studying.

**Conclusions:**

Both female and male medical students have identified that quarantine has caused them to feel emotionally detached from family, fellows, and friends and decrease their overall work performance and study period. The findings also show that one fourth of the medical students who participated in this study felt disheartened during the quarantine period. The long-term quarantine due to COVID-19 pandemics may causes further worsening in the psychological and learning behaviors of these medical students.

## INTRODUCTION

The novel Severe Acute Respiratory Syndrome Coronavirus 2 (SARS-CoV-2) also called COVID-19 pandemic emerged from Wuhan, China, and has spread all over the world causing huge threats to human health and lives.^1^ The COVID-19 has diverse epidemiological and biological characteristics, making it more contagious than earlier pandemics such as SARS-CoV and MERS-CoV.^2^ The severe contagious nature of COVID-19 developed a unique threatening situation in the entire world. Globally approximately 3.9 billion people are quarantined in their homes.^3^

The quarantine or physical isolation polices are implemented by many countries to contain their people, minimize the spread of disease, and control the contamination of infection. The quarantine included short to medium-term lockdowns, voluntary home restriction, cancellation of social and public events, and travel restrictions.^4^

In COVID-19 pandemic, medical fraternity including physicians, nurses, paramedical staff and medical students are at high risk as they are performing their duties in hospitals, causing increased stress^5^ in these unprepared situations.^6^ The current quarantine conditions have shut down schools and universities and have suspended face-to-face teaching and learning sessions. This has affected the physiological phases of lives, and induced many individual and collective health, socioeconomic, psychological and educational concerns. The aim of the present study was to investigate the impact of two weeks quarantine on medical students’ mental wellbeing and learning behaviors during this period of social isolation.

## METHODS

### Study design and settings

The present questionnaire based observational study was conducted in the Department of Physiology, College of Medicine, King Saud University during the period April 2020.

### Selection of the students

The details of the medical students, including names, addresses, phone numbers, email addresses age, gender and levels of the medical school education was obtained from the Vice Dean Office and student council, College of Medicine, King Saud University, Riyadh. The selection of students was random, and student’s information was kept confidential. We employed a power formula to calculate the sample size.^7^ The sample size was obtained by using simple random sampling. In this study, undergraduate medical students were invited to participate from medical school of King Saud University, Riyadh, Saudi Arabia.

### Questionnaire

The questionnaire was designed based on the information taken from earlier studies and edited to suit the objectives.^5^,^8^ A pilot study was conducted to test the validity of the questionnaire. The final version of the questionnaire contained 20 questions divided into three main parts. The first part was about demographic features; the second was designed to assess psychological wellbeing and the third section was based on learning behaviour queries.

Online questionnaires were distributed among the medical students through emails to collect the required information. We used a questionnaire with Five Point Likert Scale to collect the information. The Five Point Likert Scale is a well-known and highly accepted scale that can help to assess the qualitative magnitude of targeted outcomes.^9^,^10^

The survey included an opening page for informed consent and an opening paragraph that asks whether or not they agree to participate in the study. The participants were given the option to choose whether to participate or not by clicking on the box corresponding to their responses. Students who opted to participate were led to a series of questions. No reward of any kind was given to the participants. The collected information was kept completely confidential. The electronic questionnaire was distributed among the 625 medical students through their emails, and 530 (84.8%) of the medical students responded. Thus, the total number of participants was 530, and out of this total, 294 (55.47%) were female and 236 (44.53%) male.

### Statistical Analysis

The findings were analyzed by using (SPSS) software version 22.0 for Microsoft windows. The numbers and percentages were calculated. A p-value <0.05 was considered to be significant.

### Ethical Considerations

Ethical approval was obtained from the Institutional Review Board, College of Medicine, King Saud University, Riyadh, Saudi Arabia (Ref #: 20/0308/IRB).

## RESULTS

The survey questionnaire was distributed among the 625 medical students through their emails. The total number of participants who responded were 530 (84.8%); 294 (55.47%) were female and 236 (44.53 %) were male. The mean age for females was 21.2 years, and for males was 22.56 years (Table-I). The survey questionnaire consisted of total 20 items; 12 items were related to psychological wellbeing allied queries and 08 items were on learning behaviors.

**Table-I:** Medical students anthropometric characteristics (n=530).

Sociodemographic Characteristics	Number and Percentage
*Gender*	

Male	236 (44.52%)
Female	294 (55.48%)

*Age (Year)*	

Male	22.56 ±1.64 year
Female	21.2 ±1.59 year

*Marital Status*	

Single	522 (98.50%)
Married	08 (1.50%)

*Level of study (College year)*	

Year 1	40 (7.54%)
Year 2	112 (21.13%)
Year 3	91 (17.16%)
Year 4	71 (13.39%)
Year 5	216 (40.75%)

Table-II shows twelve stress allied responses were enquired from the medical students. Table-II. One hundred twenty seven (43.2%) female and 107 (45.34%) male strongly agreed for “a sense of being emotionally detached from family, fellows and friends”. The combined percentage of these students was 234 (44.15%). While enquiring about “depressed during this quarantine period” 66 (22.45%) female and 59 (25.00%) male, about one fourth of the total number medical students felt depressed during the quarantine period of two weeks (Table-II).

**Table-II:** Medical students feedback about stress allied queries (n=530).

Survey statement	Strongly agree & Agree		Neutral		Strongly disagree & Disagree		Parametric P-value

	Female	Male	Female	Male	Female	Male	
Do you have frequent thoughts of being infected during this pandemic?	98 (33.33)	43 (18.22)	96 (32.65)	57 (24.15)	100 (34.01)	136 (57.63)	<.001
Have you felt depressed during this quarantine?	66 (22.45)	59 (25)	62 (21.09)	69 (29.24)	166 (56.46)	108 (45.76)	0.034
Have you felt hopeless, exhausted or emotionally unresponsive during this quarantine?	116 (39.46)	86 (36.44)	40 (13.61)	43 (18.22)	138 (46.94)	107 (45.34)	0.339
Have you noticed a reduction in your awareness or feeling of being in a confusion?	103 (35.03)	85 (36.02)	51 (17.35)	65 (27.54)	140 (47.62)	86 (36.44)	0.006
Have you felt a sense of being emotionally detached from family, friends, etc.?	127 (43.2)	107 (45.34)	64 (21.77)	53 (22.46)	103 (35.03)	76 (32.2)	0.789
Did you invest more time on reading or watching COVID-19 related information?	113 (38.44)	47 (19.92)	76 (25.85)	81 (34.32)	105 (35.71)	108 (45.76)	0.001
Do you have anxiety dealing with febrile patients?	78 (26.53)	89 (37.71)	109 (37.07)	72 (30.51)	107 (36.39)	75 (31.78)	0.022
Have you been anxious or having insomnia during this quarantine?	127 (43.2)	79 (33.47)	47 (15.99)	37 (15.68)	120 (40.82)	120 (50.85)	0.047
Have you episode of indecisiveness or poor concentration during this quarantine?	132 (44.9)	80 (33.9)	55 (18.71)	41 (17.37)	107 (36.39)	115 (48.73)	0.012
Have you been afraid of going home because there is a possibility of infecting your family?	110 (37.41)	90 (38.14)	51 (17.35)	61 (25.85)	133 (45.24)	85 (36.02)	0.027
Are you having a lack of motor coordination?	56 (19.05)	73 (30.93)	47 (15.99)	18 (7.63)	191 (64.97)	145 (61.44)	0.001
Are you feeling a slowness in execution of movement?	79 (26.87)	64 (27.12)	36 (12.24)	51 (21.61)	179 (60.88)	121 (51.27)	0.010

While inquiring about “feelings of hopeless, exhausted or emotionally unresponsive during this quarantine period” 116 (39.46) female and 86 (36.44%) male agreed that they felt hopeless, exhausted, or emotionally unresponsive during the quarantine period. The total number of these students was 202 (38.11%).

A total of 08 items related to the quarantine impact on students’ learning behaviors used are shown in Table-III. 165 (56.12%) of the females, and 103 (43.64%) of the males strongly agreed with a statement describing “deterioration in work performance and studying subjects contents.” It was noted that 181 (61.56%) of the females and 117 (49.58%) the males had experienced “decreased in overall study period”. The combined percentage of students who felt that their study period was decreased was 298 (56.22%). While enquiring about “concentrating on the studies” 83 (28.23%), of the female medical students and 89 (37.71%) of the male students having difficulty in performing mental calculations (Table-III).

**Table-III:** Medical student’s feedback on the impact of quarantine on students’ learning behaviors (n=530).

Survey statement	Strongly agree and Agree		Neutral		Strongly disagree and Disagree		Parametric P-value
	Female	Male	Female	Male	Female	Male	
Have you noticed deterioration in your work performance/studying?	165 (56.12)	103 (43.64)	45 (15.31)	47 (19.92)	84 (28.57)	86 (36.44)	0.017
Do you remember your subject’s contents appropriately?	69 (23.47)	94 (39.83)	140 (47.62)	91 (38.56)	85 (28.91)	51 (21.61)	.001
Are you appropriately concentrating on your studies?	83 (28.23)	89 (37.71)	90 (30.61)	40 (16.95)	121 (41.16)	107 (45.34)	.001
Are you having a difficulty in performing two task simultaneously	134 (45.58)	61 (25.85)	59 (20.07)	98 (41.53)	101 (34.35)	77 (32.63)	.001
Are you having a difficulty in performing mental calculation?	63 (21.43)	43 (18.22)	76 (25.85)	48 (20.34)	155 (52.72)	145 (61.44)	0.127
Are you having a difficulty in recalling recent information?	92 (31.29)	48 (20.34)	54 (18.37)	60 (25.42)	148 (50.34)	128 (54.24)	0.009
Are you having a difficulty in recalling old information?	91 (30.95)	37 (15.68)	78 (26.53)	95 (40.25)	125 (42.52)	104 (44.07)	<.001
Are the hours of study increased or decreased?	181 (61.56)	〉117 (49.58)	51 (17.35) Same	47 (19.92) Same	62 (21.09)	72 (30.51)	0.015

## DISCUSSION

Health is a state of physical, mental, and social wellbeing and not merely the absence of disease or infirmity.^11^ Human health and wellbeing are discretely rooted in being surrounded by a functioning society. The COVID-19 outbreak has induced a global public and mental health crisis as well as a huge psycho-social experiment. We have noticed that quarantine due to the COVID-19 pandemic has caused stress and changes in the learning behaviors of medical students. Both male and female participants exhibited deterioration in their study and work performance.

With the current worldwide trend of quarantine and isolation due to Covid-19, this deterioration in mood seems to have occurred sooner than one might have expected. Hawryluck et al^5^ conducted a study on psychological effects of quarantine, and reported that quarantined persons exhibited a high prevalence of psychological distress: posttraumatic stress disorder (28.9%) and depression (31.2%).

The world has witnessed that because of the coronavirus disease outbreak in December 2019 many countries have been asking their residents to isolate themselves at home or in quarantine premises. For example, authorities in the Kingdom of Saudi Arabia reacted to the pandemic as a responsible nation, and on March 25, 2020 decided to enact a lock-down on capital city Riyadh as a part of the fight against Covid-19 outbreak. On Thursday, 26 March, the Saudi authorities imposed a total lockdown in the city Riyadh, along with Makkah and Medina, Islam’s two holiest cities, as part of tighter constraints to counter the virus. During the current COVID-19 pandemic, with the current lockdown situation, medical students in Saudi Arabia are living in a quarantine inside their residence facilities.

More recently, Röhr *et al.*,^12^ has demonstrated that quarantine measures during COVID-19 outbreaks have severe negative consequences for mental health. In another study, Lei *et al.*,^13^ identified high prevalence of anxiety and depression among people who quarantined during COVID-19 outbreak in southwestern China. Similarly in the present study, we found that 125 (23.5%) medical students felt disheartened and depressed. The most probable reasons for negative consequences for mental health during quarantine are that people considered it an unpleasant experience, because of having to depart from fellows, friends and family, losing the ability to move about freely, experiencing doubts about the spread of disease, and developing intense feelings and reactions.

The quarantine phase is a complex intervention within the jurisdiction of public health.^14^ Our results demonstrate two important characteristics related to the COVID-19 quarantine, namely an increase in stress and an increase in dysfunctional learning behaviors. Although, medical students believe that quarantine has not much affected their learning and psychological wellbeing, the students whom we interviewed stated that quarantine has caused them to feel emotionally detached from family, fellows and friends, and has led to a decrease in overall work performance and duration of study.

In the past, the quarantine compliance rate has been positive, studies have indicated high levels of acceptance of quarantine among samples of Toronto-area residents (97%) and US citizens (93%) during a SARS 1 pandemic quarantine.^15^,^16^ In the current study, a good number of responses were also recorded where participants have a difference of opinion. The female and male medical students showed a sense of being emotionally detached from family, fellow professionals and friends, feel depressed and decreased in their overall study work performance and study period.

The literature had consistently reported differences occurring concerning stress among males and females. This is constant with preceding research indicating that female physicians are more likely to be emotionally fatigued at the commencement of exhaustion, compared to male physicians.^17^ Similarly, female students in the present study were found to be motivated in handling their learning, especially when it came to performing two tasks simultaneously. Brooks *et al.*,^18^ reported negative psychological effects including post-traumatic stress symptoms, confusion, and anger due to the COVID-19 outbreak quarantine. The authors concluded that during a long duration quarantine, fear, frustration, inadequate information and financial loss are the main causes of negative psychological impact. Similarly, the present study findings suggest that the reported alterations in attitude and mood may be due to the threatening situation of the COVID-19 pandemic. Medical students, in particular, may feel differently once this situation is over, and explicit research should explore such differences.

## CONCLUSION

Both female and male medical students have identified that quarantine has caused them to feel emotionally detached from family, fellows, and friends, and has decreased their overall work performance and study period. The findings also show that one fourth of the medical students who participated in this study felt depressed during the quarantine period of two weeks. The long-term quarantine due to COVID-19 pandemics may cause further worsening in the psychological and learning behaviors of these medical students. The results from this study might provide to health policymakers evidence about the psychological and emotional influences of quarantine on medical students so that they might produce an important guideline for managing the medical students’ quarantine. Indeed, we believe that the current results indicate the need for supplementary mental wellbeing interventions for everyone while they are quarantined. This goal could be achieved by using a healthy diet, exercise at home, proper sleep and engaging in healthy physical, intellectual and educational activities. In situations where quarantine is deemed necessary, the policy makers should quarantine individuals for no longer than a required period.

### Authors’ Contributions

**SAM:** Research conceptualization, supervision, manuscript writing.

**AAA, AAA, KS,** Methodology, literature review, data collection and data analysis.

**DCK:** Manuscript writing. All authors have read and agreed to the published version of the manuscript.
